# Pre-pandemic Physical Function and Social Network in Relation to COVID-19-Associated Depressive Burden in Older Adults in Sweden

**DOI:** 10.1093/geroni/igac041

**Published:** 2022-06-09

**Authors:** Federico Triolo, Marguerita Saadeh, Linnea Sjöberg, Laura Fratiglioni, Anna-Karin Welmer, Amaia Calderón-Larrañaga, Serhiy Dekhtyar

**Affiliations:** Aging Research Center, Department of Neurobiology, Care Sciences and Society, Karolinska Institutet and Stockholm University, Solna, Sweden; Aging Research Center, Department of Neurobiology, Care Sciences and Society, Karolinska Institutet and Stockholm University, Solna, Sweden; Aging Research Center, Department of Neurobiology, Care Sciences and Society, Karolinska Institutet and Stockholm University, Solna, Sweden; Aging Research Center, Department of Neurobiology, Care Sciences and Society, Karolinska Institutet and Stockholm University, Solna, Sweden; Stockholm Gerontology Research Center, Stockholm, Sweden; Aging Research Center, Department of Neurobiology, Care Sciences and Society, Karolinska Institutet and Stockholm University, Solna, Sweden; Division of Physiotherapy, Department of Neurobiology, Care Sciences and Society, Karolinska Institutet, Stockholm, Sweden; Aging Research Center, Department of Neurobiology, Care Sciences and Society, Karolinska Institutet and Stockholm University, Solna, Sweden; Stockholm Gerontology Research Center, Stockholm, Sweden; Aging Research Center, Department of Neurobiology, Care Sciences and Society, Karolinska Institutet and Stockholm University, Solna, Sweden

**Keywords:** Aging, Coronavirus pandemic, Depression, Prevention, Resilience

## Abstract

**Background and Objectives:**

The coronavirus disease 2019 (COVID-19) pandemic, as well as the measures intended to limit its spread, have likely affected older adults’ depressive burden. Good physical functioning and a rich social network may benefit older adults’ mental health. We examined whether pre-pandemic physical functioning and social network were associated with depressive burden during the first wave of the COVID-19 pandemic in Stockholm, Sweden.

**Research Design and Methods:**

A telephone assessment of depressive burden using the symptoms of sadness, anxiety, worrying, reduced sleep, and reduced appetite was conducted in May–September 2020 in 930 older adults from the Swedish National Study on Aging and Care in Kungsholmen (SNAC-K), an ongoing population-based study. Objective measures of gait speed, muscle strength, and balance; and self-reports of social connections and support were collected in 2016–2019. Logistic models were adjusted for sociodemographic, clinical, lifestyle, and pandemic-related factors (loneliness, change in physical and social engagement, and experience of death due to COVID-19).

**Results:**

Only good muscle strength (odds ratio [OR]: 0.53; 95% confidence interval [CI]: 0.32–0.85; ref: poor strength, ≥17 s) and rich social support (OR: 0.67; 95% CI: 0.45–0.99; ref: poor support) exhibited an independent association with depressive burden, even after accounting for pandemic-related factors. A combination of good muscle strength and rich social support were associated with the greatest reduction in depressive burden (OR: 0.35; 95% CI: 0.18–0.66; ref: poor social support and poor muscle strength).

**Discussion and Implications:**

Prepandemic social support and muscle strength could supply older adults with resilience against the depressive burden associated with the COVID-19 pandemic.

**Translational Significance:** When the care system struggles to meet the complex needs of older adults during moments of severe crises, as that brought by the coronavirus disease 2019 (COVID-19) pandemic, it is important to identify modifiable aspects of older adults’ pre-pandemic lives that could supply them with resilience against the mental adversity associated with the pandemic. Our findings highlight physical functioning, particularly muscle strength, as well as a social network, and particularly social support, as possible modifiable sources of resilience against the depressive burden associated with the COVID-19 pandemic. Geriatric practitioners should continue promoting physical and social engagement among older adults, particularly during the COVID-19 pandemic.

Ever since the first confirmed case was reported in Sweden on January 31, 2020, coronavirus disease 2019 (COVID-19) has had a profound impact on all aspects of the Swedish society. In contrast to many other countries, Sweden pursued a less restrictive approach and largely avoided mandatory lockdowns during the first wave of the pandemic in April–August 2020. Instead, individuals were requested to exercise personal responsibility to protect themselves and others (Calderón-Larrañaga et al., 2020). At the same time, recommendations to minimize the spread of SARS-CoV-2 were considerably more stringent for persons aged 70 years or older, with possible adverse consequences for their mental well-being (Public Health Agency of Sweden, 2021). Specifically, the ban on visits to old age care facilities was put in place, whereas older adults living at home were strongly advised against physical contact with family and friends.

Initial findings suggest that the mental health impact of the first wave of the pandemic in Swedish older adults has been considerable. A survey of over 1,200 individuals aged 68–103, has found that nearly half of them experienced some form of psychological burden (feelings of worry, stress, or loneliness) during March–August 2020 (Beridze et al., 2022). This burden was likely exacerbated by the suspension of nonacute in-person visits to mental health care facilities for those aged 70 or older. Distance- and outpatient care were meant to compensate for this reduction, although the rates of outpatient visits remained unchanged, while the number of acute patients in the geriatric psychiatry departments actually dropped by one-quarter in March–July 2020. As a result, some have argued that older adults’ mental health problems that emerged during the first wave of the pandemic may not have been adequately addressed by the Swedish health care system (Skoog, 2020).

Because the care system can understandably struggle to meet the complex needs of older adults during moments of severe crises, as that brought by the COVID-19 pandemic, it is worth identifying modifiable aspects of older adults’ pre-pandemic lives that could supply them with resilience against the mental adversity associated with the pandemic, as well as the lockdown measures intended to limit its spread. Prepandemic physical functioning and social network could be such factors.

Starting with the former, low physical functioning and mental ill-health are closely linked, and share several risk and pathogenic factors, including oxidative stress and inflammation (Alexopoulos & Morimoto, 2011; Jimenez-Fernandez et al., 2015). Furthermore, reduced physical functioning can be a marker of premature deterioration of the nervous system (Grande et al., 2020; Veronese et al., 2017). When it comes to a social network, social connectedness and social support may reduce depression by limiting social isolation, or by activating positive affective states that instill a sense of purpose and self-worth, as well as promote coping strategies, particularly when faced with stressful events (Kawachi & Berkman, 2001; Triolo et al., 2020). Socially integrated lives can also help forestall cognitive decline, which is strongly associated with depression in old age (Fratiglioni et al., 2020). There is a further possibility of a vicious cycle of damage, whereby declining physical functioning and social disengagement synergistically reinforce one another, translating into a markedly worsened aging trajectories and increased risk of depression (Calderón-Larrañaga et al., 2021; Veronese et al., 2017). While tentative evidence suggests that social networks and physical functioning have become highly relevant for mental well-being during the COVID-19 pandemic (Jacob et al., 2020; Kua et al., 2022; Pieh et al., 2020), it is mostly based on cross-sectional reports that used contemporaneous assessments of depressive burden, social-, and physical factors, which may be subject to reverse causation. Furthermore, few studies have sought to determine specifically which physical and social domains were most associated with psychological resilience.

In this study, our aim was to examine the association of pre-pandemic levels of physical functioning and social network with older adults’ depressive burden experienced during the first wave of the COVID-19 pandemic in March–September 2020 in Stockholm, Sweden. We do so by embedding a telephone interview aimed at monitoring preventive behaviors and health consequences of the COVID-19 pandemic within an ongoing population-based study of older adults. Importantly, we introduce a temporal gap between physical function and social network on one side and depressive burden on another, while accounting for pandemic-associated changes in physical activity, social contact, loneliness, and bereavement. This enables us to disentangle the independent contribution of several domains of pre-pandemic physical functioning and social network to the depressive burden elicited during the first COVID-19 wave in Stockholm.

## Method

### Study Population

This study examines data from the Swedish National Study of Aging and Care in Kungsholmen (SNAC-K), a longitudinal cohort of older adults residing in central Stockholm established in 2001. Individuals were randomly sampled within 11 age-cohort strata (60, 66, 72, 78, 81, 84, 87, 90, 93, 96, and ≥99 years), with a total of 3,363 older adults participating in the baseline assessment in 2001–2004 (73% participation rate). Repeated assessments were conducted every 3 years (for the older age groups [≥78 years]) or 6 years (for the younger age groups [<78 years]). New age cohorts were further added over the follow up to counteract attrition (please see www.snac-k.se for more details).

During the COVID-19 outbreak, all individuals (*n* = 1,442) scheduled to be examined during the 2019–2021 wave of data collection (wave 7) were considered for participation in a telephone interview aimed at monitoring preventive behaviors and health consequences of the COVID-19 pandemic. Participants with previous cognitive or major physical impairment, nursing home residents, as well as individuals deceased since the 2016–2019 wave (*n* = 103) were excluded from the data collection, resulting in an eligible interview sample of 1,339. As 108 individuals could not be reached or refused to be interviewed, 1,231 participants were assessed between May and September 2020 (participation rate 91.9%). After ensuring that these participants had also been examined in the 2016–2019 wave (which we considered as baseline for the purpose of this study), the population eligible for analysis amounted to 930 participants (age cohorts 66, 81, 84, 87, 90, 93, and ≥96 years). Accounting for missing data on the depressive burden, key exposures, and covariates resulted in the analyzed sample ranging from *n* = 820 to *n* = 703, depending on the model (actual sample sizes provided alongside model estimates).

All phases of the SNAC-K study, as well as the specific COVID-19 data collection (dnr: 2020-02497) received approval from the Regional Ethics Review Board in Stockholm. Each participant, or their next of kin (in case of cognitive impairment), provided informed written consent.

### Depressive Burden

Depressive symptomatology during the COVID-19 outbreak was examined via the telephone using five items selected from the Comprehensive Psychopathological Rating Scale (CPRS): sadness, anxiety, worrying, reduced sleep, and reduced appetite. CPRS is a semistructured tool assessing the presence and severity of multiple psychiatric signs and symptoms on a zero- (no depressive burden) to-six- (severe depressive burden) scale. A score of two designates clinically relevant symptomatology (Åsberg et al., 1978). As the aim was to capture symptomatology specific to the first COVID-19 outbreak in Sweden, the questions instructed participants to explicitly reflect on symptoms occurring since March 2020. To operationalize depressive burden, we first summed the scores of the depressive symptoms (range 0–30), and then dichotomized them by the median into “low” (<7) and “moderate/high” (≥7). If a participant was missing any symptom (*n* = 22), the depressive burden was not calculated.

### Physical Functioning

Physical function prior to the COVID-19 outbreak was objectively assessed using three tests administered during the 2016–2019 data collection: gait speed, chair stand, and balance.

#### Gait speed

Walking speed was measured as the time required to walk 6 m or (when walking was impaired or if testing space was limited, e.g., in participants’ residence), 2.4 m at self-selected pace. Measurements were expressed as meters per second (m/s). Following an agreed-upon clinical cutoff (Van Kan et al., 2009), participants with gait speed above 0.8 m/s were defined as having “fast walking speed,” while individuals below 0.8 m/s and those unable to carry out the test due to severe physical limitations had “slow walking speed.”

#### Muscle strength

The chair stand test was carried out by having participants raise up from a seated position five times as fast as possible, without the aid of their arms. The time in seconds needed to perform the test was recorded, and, in accordance with an established clinical threshold (Ward et al., 2015), a score below 17 s was used to define “good muscle strength.” Participants unable to execute the test were deemed to have “poor muscle strength.”

#### Balance

Balance was assessed by measuring the maximum time (up to 60 s) participants could stand on one leg with their eyes open. Both legs were tested, and the best score was used in the analyses. Individuals able to stand for 5 s or more were categorized as having “good balance.” Participants unable to perform the task were also considered as having “poor balance” (Vellas et al., 1997).

### Social Network

Information on the social network prior to the pandemic was also collected from the 2016 to 2019 data collection wave. Two measures of the social network were used: social connections and social support, which reflect network structure and function, respectively (Holt-Lunstad et al., 2017).

#### Social connections

Social connections comprised information on marital status, living arrangement, social network size, parenthood, friendships, and frequency of direct contact with parents, children, neighbors, and friends (Online Supplementary Material).

#### Social support

Social support assessment closely followed the questionnaire used in (Hanson et al., 1997) and incorporated information on satisfaction with social contacts with family and friends, perceived material and psychological support, sense of affinity with neighbors and relatives, and belonging to a circle of friends (Online Supplementary Material).

Using the procedure suggested by (Cornwell & Waite, 2009), we generated the indices of social connections and social support by z-transforming each individual measure first, and then averaging them separately across the domains of connections and support. In the analysis, the two indices were operationalized according to the tertile. The highest two tertiles identified rich social connections/support, while the lowest tertile designated low social connections/support. Cronbach’s alphas for social connections and social support were 0.90 and 0.88, respectively.

### Covariates

#### Prepandemic factors

Education was assessed as the highest degree attained and was dichotomized as high school or below versus university. Multimorbidity burden was assessed with the presence of at least two chronic diseases using a previously described operationalization building on information from health registers and clinical examinations (Calderón-Larrañaga et al., 2017). Prepandemic depressive syndrome was defined as a Montgomery-Åsberg Depression Rating Scale (MADRS) score >6 in the 2016–2019 wave (Snaith et al., 1986). MADRS is a depression rating scale obtained from ten selected depressive items from CPRS (Montgomery & Åsberg, 1979). Prepandemic levels of smoking (“current” vs “never or ever”) and alcohol use (“non/moderate drinkers” vs “heavy drinker”) were also considered.

#### Pandemic-related factors

As part of the COVID-19 telephone interview, we also gathered information on pandemic-related factors for which we accounted in additional analyses. Loneliness during the outbreak was measured with the University of California Los Angeles Three-Item Loneliness Scale, which sums three components: lack of company, feeling left out, and feeling isolated (Hughes et al., 2004). Loneliness was included in the models as a continuous variable (range: 3–9), with higher values representing the greater experience of loneliness (correlation with depressive burden *r* = 0.37). Experience of death of a relative due to COVID-19 was recorded as a binary variable (yes/no). A self-reported change in physical activity during the pandemic was also considered (operationalized as reduced light or intense physical activity vs no reduction). Finally, a change in interactions with family and friends (both in-person and through phone/video) was also collected (operationalized as reduction/no reduction).

### Statistical Analyses

First, we assessed the associations of pre-pandemic levels of physical function and social network with the presence of depressive burden using separate sets of logistic models: one for the three physical function measures (gait speed, balance, and muscle strength), one for the two social network domains (connections and support). We adopted a sequential model building strategy: Model 1: sociodemographic factors (age, sex, and education); Model 2: Model 1 + clinical factors (multimorbidity and previous depressive syndrome); Model 3: Model 2 + lifestyle behaviors (alcohol use and smoking); Model 4: Model 3 + pandemic-related factors (loneliness, death of relative due to COVID-19, change in physical activity, and change in social interactions). We subsequently assessed the independent contribution of physical functioning and social network to depressive burden in models that incorporated both factors. Finally, we assessed the joint affect of the two domains in combined analyses using an indicator variable constructed to designate four possible combinations of physical function and social network (from low/low to high/high) using factors that were individually associated with depressive burden based on the previous analysis. STATA 17 was used to carry out all analyses.

## Results

Table 1 reports the descriptive characteristics of the full analytical sample, as well as according to the presence of depressive burden during the COVID-19 outbreak. Out of 930 participants, 35% presented a depressive burden, with the prevalence of single symptoms being: reduced sleep (26%), worrying (18%), anxiety (12%), sadness (18%), and reduced appetite (5%; all prevalence estimates use a symptom cutoff score of ≥2 for each item of the CPRS).

**Table 1.** Baseline Characteristics of the Study Population According to Depressive Burden Experienced During the First Wave of COVID-19 Pandemic in May–September 2020

Characteristics	Eligible study population	Depressive burden^a^		
		Low	Moderate/high	
	*n* = 930	*n* = 592	*n* = 316	*p* Value
Factors assessed in 2016–2019				
Age (during phone interview), mean (*SD*)	74.7 (9.45)	74.3 (9.3)	75.3 (9.5)	.12
Sex, female, %	64	58	77	<.01
Education, university, %	57	58	55	.37
Civil status, married, %	52	58	41	<.01
Multimorbidity, 2+ diseases, %	92	89	96	<.01
Previous depression, MADRS > 6, %	10	5	19	<.01
Smoking, current, %	7	8	5	.17
Alcohol use, heavy, %	20	18	24	<.05
Walking speed, fast (≥0.8 m/s), %	84	86	81	.06
Muscle strength (chair stand test), good (<17 s), %	79	83	72	<.01
Balance (one-leg test), good (>5 s), %	66	70	61	<.05
Social connections, rich, %	67	68	64	.20
Social support, rich, %	67	70	62	<.05
Factors assessed during COVID-19 phone interview^b^				
COVID-related loneliness, % >median	33	22	53	<.01
COVID-related reduction in social interaction, %	20	18	24	<.05
COVID-related reduction in physical activity, %	48	42	58	<.01
Death of relative due to COVID-19, %	4.88	4.9	4.8	.94

*Notes:* MADRS = Montgomery-Åsberg Depression Rating Scale; *SD* = standard deviation; COVID-19 = coronavirus disease 2019.

^a^Statistics according to depressive burden were calculated on the sample of persons without missing data in assessed symptoms: 930 (eligible population)−22 (missing at least one symptom) = 908 (nonmissing depressive burden). Depressive burden is dichotomized according to the median depressive score as “low” (<7) and “moderate/high” (≥7).

^b^COVID-19 phone interview took place in May–September 2020.

The associations of pre-pandemic physical function domains and social network components with depressive burden during the pandemic are explored in Table 2. Of the three pre-pandemic physical function measures, only good chair stand performance (a proxy of muscle strength) exhibited an association with lower odds of depressive burden during the first COVID-19 wave (odds ratio [OR]: 0.53; 95% confidence interval [CI]: 0.32–0.87). While the magnitude of the association was progressively weakened upon adjustment for covariates (Models 1–3), further consideration of pandemic-concurrent loneliness (i.e., change in physical- and social engagement, as well as experience of close death due to COVID-19) resulted in the strengthening of the point estimate (OR [good chair stand performance]: 0.47; 95% CI: 0.26–0.84; Model 4).

**Table 2.** Associations Between Prepandemic Physical Function and Social Network With Depressive Burden During the First Wave of the COVID-19 Pandemic in May–September 2020

Exposures	Model 1	Model 2	Model 3	Model 4
	Age and sex education	Model 1 + MM and previous depression	Model 2 + smoking and alcohol	Model 3 + COVID-19 factors^a^
**Contribution of physical function dimensions to depressive burden** ^b^	*n* = 814	*n* = 814	*n* = 784	*n* = 704
Fast walking speed (ref: <0.8 m/s)	0.92	0.97	0.92	0.86
	(0.56–1.52)	(0.58–1.61)	(0.55–1.55)	(0.47–1.56)
Good balance (ref: <5 s)	0.93	1.03	1.00	1.29
	(0.59–1.45)	(0.65–1.62)	(0.62–1.60)	(0.75–2.21)
Good chair stand (ref: ≥17 s)	0.53	0.56	0.58	0.47
	(0.32–0.87)	(0.34–0.92)	(0.35–0.97)	(0.26–0.84)
**Contribution of social network dimensions to depressive burden** ^b^	*n* = 820	*n* = 820	*n* = 787	*n* = 707
Rich social support (ref: poor support)	0.63	0.70	0.66	0.62
	(0.45–0.88)	(0.49–0.99)	(0.46–0.95)	(0.41–0.93)
Rich social connection (ref. poor connection)	0.99	1.01	0.96	1.03
	(0.71–1.38)	(0.72–1.41)	(0.68–1.36)	(0.69–1.54)
**Independent contribution (mutual adjustment) of chair stand and social support to depressive burden** ^b^	*n* = 813	*n* = 813	*n* = 781	*n* = 703
Good chair stand (ref: ≥17 s)	0.55	0.61	0.61	0.53
	(0.37–0.83)	(0.41–0.93)	(0.41–0.94)	(0.32–0.85)
Rich social support (ref: poor support)	0.68	0.74	0.69	0.67
	(0.49–0.94)	(0.53–1.03)	(0.49–0.98)	(0.45–0.99)

*Notes:* MM = multimorbidity (2+ chronic conditions); COVID-19 = coronavirus disease 2019. Estimates come from several logistic models.

^a^COVID-19 factors refer to reduction in physical activity and social interaction, loneliness, and bereavement. All assessed as change from March, 2020.

^b^Outcome: moderate or high depressive burden (vs low).

Of the two pre-pandemic social network domains, only rich social support was associated with lower odds of depressive burden (OR: 0.63; 95% CI: 0.45–0.88); an association which weakened as pre-pandemic disease burden, history of depression, and lifestyle behaviors were considered (Models 1–3) but strengthened in Model 4 that further incorporated a host of pandemic-concurrent factors (OR [high support] 0.62; 95% CI: 0.41–0.93).

Having identified pre-pandemic chair stand performance and social support as the only physical and social correlates of depressive burden during the COVID-19 pandemic, we proceeded to assess their independent contributions in mutually adjusted models (Table 2, bottom rows). While both retained their association with lowered odds of depressive burden, the point estimate for chair stand was consistently greater than for social support (OR [Model 3 with full pre-pandemic covariate adjustment]: 0.61; 95% CI: 0.41–0.94 [good chair stand] and 0.69; 95% CI: 0.49–0.98 [rich social support]). Further consideration of pandemic-concurrent factors strengthened both estimates: OR 0.53; 95% CI: 0.32–0.85 (good chair stand) and 0.67; 95% CI: 0.45–0.99 (rich social support). We did not observe statistically significant interactions between either chair stand or social support with age, sex, or education for depressive symptoms.

**Figure 1.**
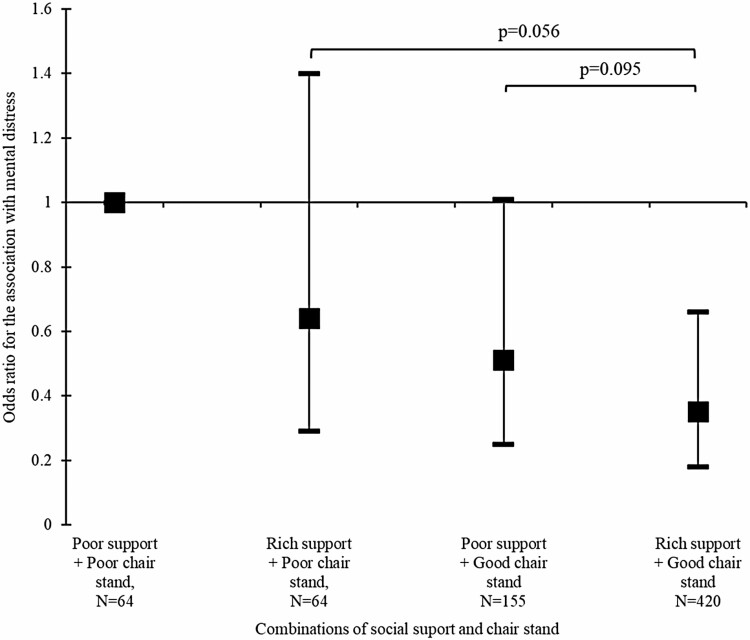
Joint influence of pre-pandemic levels of chair stand test and social support for the odds of experiencing moderate or high depressive burden during the first wave of COVID-19 pandemic in May–September 2020. COVID-19 = coronavirus disease 2019. *Notes:* Estimated from a logistic regression adjusted for age, sex, education, multimorbidity, previous depression, smoking, alcohol, and COVID-19 experiences (loneliness, change in physical and social engagement, and experience of a close death due to COVID-19). *p* Values from tests of parameter equality are presented above the confidence interval bars.

Finally, we explored the interplay of pre-pandemic chair stand performance and social support for depressive burden using a four-level indicator variable designating possible combinations of the two binary factors (multiplicative interaction *p* = .175). Figure 1 plots the estimates obtained from a fully adjusted model, incorporating both pre-pandemic and pandemic-concurrent covariates. The odds of depressive burden in the rich social support/poor chair stand group were not statistically different from the reference group (poor support/poor chair stand). In contrast, those with poor support but with good chair stand performance presented with a considerably lower odds of a depressive burden compared with the reference group (albeit marginally significant, OR = 0.51; 95% CI: 0.25–1.01; *p* = .052). Finally, the group defined by both rich social support and good chair stand exhibited statistically significantly lower odds of depressive burden relative to the reference group (OR: 0.35; 95% CI: 0.18–0.66). Postestimation tests of parameter equality suggested that in addition to being statistically different from the reference group, the estimates for the rich support/good chair stand group were marginally different from the rich support/poor chair stand group (*p* = .056), while the difference with the poor support/good chair stand group was somewhat less clear (*p* = .095).

## Discussion

In this epidemiological study, we combined pre-pandemic assessments of physical functioning and social network obtained as part of the ongoing SNAC-K population-based study, with reports of depressive burden during the first wave of the COVID-19 pandemic (May–September 2020) in Stockholm, Sweden. This enabled us to investigate if modifiable aspects of older adults’ pre-pandemic physical function and social network could supply them with resilience against the mental adversity associated with the pandemic, as well as the lockdown measures intended to limit its spread.

Our results revealed that only chair stand performance (i.e., a proxy of muscle strength) from among the physical function measures, and only social support from among the social network domains, exhibited an independent association with depressive burden during the first COVID-19 wave in Stockholm. The association with depressive burden appeared greater for chair stand than for social support, although their confidence intervals largely overlapped. A combination of good chair stand performance and rich social support was associated with the greatest reduction in the odds of experiencing depressive burden, although the synergistic effect is tentative, given the overlapping confidence intervals in the joint indicator analysis.

These findings highlight physical function and social network as potential modifiable sources of resilience against mental-ill health associated with the COVID-19 pandemic. Starting with the former, physical function, has been consistently linked with anxiety (McDowell et al., 2019) and depression (Schuch et al., 2018) during nonpandemic times (Belvederi Murri et al., 2020; Wolf et al., 2021). This association appears to have been preserved during the first COVID-19 wave, suggesting how such factors may still be relevant for mental health in spite of the direct and indirect consequences of the pandemic, which we also attempted to account for in our analyses. Thus, moderate-to-vigorous physical activity was linked with mental well-being during the COVID-19 pandemic in surveys of middle-aged adults from the U.K. (Jacob et al., 2020) and Austria (Pieh et al., 2020), while short-term reduction in physical activity levels during the lockdown was associated with poorer psychological outcomes in a survey of health care workers in Singapore (Kua et al., 2022). Our results corroborate and advance these cross-sectional findings by using pre-pandemic measures of physical activity, thus ensuring that physical functioning is not a consequence of mental distress during the pandemic.

People with depression present with lowered markers of neurogenesis, as well as increased levels of inflammatory and oxidant markers (Schuch et al., 2016). Physical activity has been previously shown to attenuate hippocampal atrophy, increase neurogenesis, and help regulate the imbalance between anti- and proinflammatory, as well as oxidant markers (Loprinzi et al., 2013). Physical activity may also enhance coping efficacy, promote self-esteem, and perceptions of physical competence and subjective health (Cairney et al., 2009). Interestingly, in our study, the only measure of physical function that presented an association with depressive burden was the chair stand test, which is a widely used proxy of muscle strength (Chen et al., 2020). Why muscle strength specifically would exhibit the strongest link with depressive burden is unclear, although the recent literature suggests that muscle health may influence brain neurogenesis and plasticity (Delezie & Handschin, 2018; Erickson et al., 2013; Marinus et al., 2019). Furthermore, exercise training has been shown to affect the kynurenine metabolism and protect from stress-induced depression in transgenic mice models where skeletal muscle conditioning was isolated from other exercise components (Agudelo et al., 2014). A Cochrane review on the role of exercise in the treatment of depression has reported greater effect sizes for resistance training (which generally results in improved muscle strength), than for aerobic training (Cooney et al., 2013), which is consistent with the findings reported here, as is the finding of a seemingly reduced mental health burden during COVID-19 in elite athletes (Sokić et al., 2021). Retained muscle strength in old age may signify preserved psychological and physical resilience (Whitson et al., 2016), which could be especially relevant in older adults who are occasionally affected by sarcopenia and frailty. Notably, the beneficial role of muscle strength during the pandemic extends beyond its association with mental health, as evidenced by recent a study showing a lower likelihood of exhibiting COVID-19-like symptoms in individuals with improved muscle strength (Saadeh et al., 2022). Together, these findings underscore the role of muscle strength in resilience against the detrimental consequences of COVID-19 in older adults, although a better understanding of the underlying mechanisms is warranted.

With respect to the pre-pandemic aspects of the social network, only social support exhibited an association with depressive burden during the COVID-19 pandemic in our data. Consistent with our findings, increased levels of social support were found protective against psychological distress in a convenience sample from a general population of mainland China in early February 2020 (Yu et al., 2020). Furthermore, in a large survey, also from China, social support was found to buffer against the negative affects on mental health during the first COVID-19 wave (Li et al., 2021). Similarly, a cross-sectional study from Germany has found that greater perceived social support was associated with lower values on depressive-, anxiety-, and sleep disorder symptoms in an online survey of middle-aged participants during COVID-19’s first wave (Bauer et al., 2020). Positively functioning social networks likely instill a sense of purpose and self-worth through emotional assistance, which refers to the display of care and affection by close ones. In addition, stress due to the aging process can be buffered through informational and instrumental support provided by caregivers and social ties, which can ultimately increase psychological well-being (Thoits, 2011). By promoting healthy behaviors and adherence to recommendations aimed at limiting the COVID-19 transmission, supportive networks can help relieve stress and mitigate increased loneliness (Grey et al., 2020), further reducing the psychological distress during the pandemic.

Our study advances previous literature in several important ways. By using pre-pandemic assessments of physical function and social network we were able to mitigate the likely bidirectionality and reverse causation afflicting many cross-sectional investigations using contemporaneously collected measures of depressive burden, social-, and physical factors. Furthermore, the effects of pre-pandemic factors were estimated here net of the changing psychological, physical, and social circumstances due to the COVID-19 outbreak. In our study, nearly half of the sample reported a pandemic-associated reduction in physical activity, one third revealed increased loneliness, and one fifth indicated a reduction in social interaction with family and friends. These changing circumstances may have their own unique affect on COVID-related mental ill-health, as indicated in the United States study reporting that no longer meeting physical activity guideline recommendations during the pandemic was associated with increased depression, stress, and loneliness (Meyer et al., 2020). Notably, accounting for the contemporaneous change in psychological, social, and physical environments resulted in a strengthening of the point estimates for the two pre-pandemic factors we assessed (social support and chair stand test), underscoring the evolving nature of depressive burden during the COVID-19 pandemic in Stockholm. Future studies may consider comparing the features characterizing old-age depression before and during the pandemic.

Finally, although pre-pandemic chair stand performance and social support both retained their influence in mutually adjusted analyses, a closer inspection of their interplay underscored the relevance of physical function, which seemingly offered protection against depressive burden even in the face of poor social support. One possibility could be the exercise-specific effects on the endogenous opioid system, or its more considerable role―compared with social support―as a distractor from worries and depressing thoughts (Craft & Perna, 2004; Mikkelsen et al., 2017). This suggests that physical function, and particularly increased muscle strength, could be a particularly strong source of resilience. The lowest likelihood of depressive burden was found in those with both good muscle strength and rich social support, underscoring their synergistic influences on mental well-being, although this conclusion is tentative in the face of overlapping confidence intervals.

Strengths of this study include embedding a telephone interview assessing COVID-19-experiences within an ongoing well-characterized population-based cohort study that has amassed vast data on pre-pandemic factors. Temporal separation between exposures and outcomes, is another advantage, although this observational study cannot provide causal conclusions. Several limitations need acknowledgment. Our measurement of depressive burden during the pandemic was based on a selection of depressive symptoms that could be easily assessed over the phone, rather than on a complete rating scale. Therefore, it differed from the pre-pandemic assessment collected during SNAC-K’s wave 6 (considered as the baseline for the purpose of this study), which prevented us from examining pre–post-COVID-19 changes in depression. Additionally, assessing the symptoms by phone restricted the ability to assess observable representations of sadness, potentially increasing information bias. Furthermore, pre-pandemic physical function and social network were collected as far back as 4 years prior to the pandemic for some participants and, therefore could have changed during this period. Finally, the findings come from community-dwellers residing in a socioeconomically affluent area of Stockholm and, therefore can be poorly generalizable to other contexts.

In conclusion, these findings highlight physical functioning, particularly muscle strength, as well as a social network, and particularly social support, as possible modifiable sources of resilience against the depressive burden associated with the COVID-19 pandemic. Geriatric practitioners should continue promoting physical and social engagement among older adults, particularly during pandemic times.

## Supplementary Material

igac041_suppl_Supplementary_Material
