# Supplementary material for: Pre-pandemic Physical Function and Social Network in Relation to COVID-19-Associated Depressive Burden in Older Adults in Sweden
Source: Innov Aging. 2022 Jun 9;6(5):igac041. doi: 10.1093/geroni/igac041 (PMC9273957; doi:10.1093/geroni/igac041)
Supplement: igac041_suppl_Supplementary_Material [file igac041_suppl_supplementary_material.docx]

**SUPPLEMENTARY TEXT**

*Interview Protocol*

Instructions: Below are the questions regarding your social network. Write or check the

answers that best suit you.

| **Social Connection** | |
| --- | --- |
| *No. of living children* | 0; 1; 2; 3; 4; 5;≥6 |
| *Marital status* | Married; widowed/divorced; unmarried |
| *Living arrangement* | In an institution; alone; with someone |
| *Social network size* | |
| How many people do you feel you know well and can talk to about most things? (e.g., relatives, friends, neighbours, and/or colleagues) | None; 1-2 people; 3 people; 4-6 people; 7-9 people; 10-15 people; 16-30 people; More than 30 people; Don't know; No response; Several options checked |
| *Frequency of direct or remote contacts* | |
| How often do you meet the following in person?  1. Parents  2. Children  3. Son/daughter-in-law  4. Grandchildren  5. Siblings  6. Other relative  7. Neighbour  8. Friend | For each item:  Daily, more than twice/week  Weekly, more than twice/month  Monthly, more than 6 times/year  Quarterly, more than once/year  Less often  Never  Don't know  N/A (e.g., have no children or parent no longer alive)  No response  Several options checked |
| How often are you in touch, via telephone, letters, e-mail with the following:  1. Parents  2. Children  3. Son/daughter-in-law  4. Grandchildren  5. Siblings  6. Other relative  7. Neighbour  8. Friend | For each item:  Daily, more than twice/week  Weekly, more than twice/month  Monthly, more than 6 times/year  Quarterly, more than once/year  Less often  Never  Don't know  N/A (e.g., have no children or parent no longer alive)  No response  Several options checked |

| **Social Support** | |
| --- | --- |
| *Satisfaction with these contacts* | |
| Are you satisfied with these contacts?  1. Parents  2. Children  3. Son/Daughter-in-law  4. Grandchildren  5. Siblings  6. Other relative  7. Neighbour  8. Friend | For each item:  Yes  No  Don't know  N/A (e.g., do not have children or parents no longer alive)  No response  Several options checked |
| *Perceived material support* | |
| Could you get help from one or several people in case of illness or other practical troubles? (e.g., borrow little things, get help with repairs, get advice and information)  Do you know one or several people who could help you write an official letter or appeal a government decision? | For each item:  Yes, without a doubt  Yes, probably  No, probably not  No, not at all  Don't know  No response  Several options checked |
| *Perceived psychological support* |  |
| Do you feel that you know one or a few people who could give you proper personal/emotional support to manage the stress and troubles of life?  Do you know someone with whom you can be yourself, who accepts you for all your good and bad qualities? | For each item:  Yes, without a doubt  Yes, probably  No, probably not  No, not at all  Don't know  No response  Several options checked |
| *Sense of affinity with association members* | |
| If you are part of an association/organization, would you say you feel a strong sense of belonging to this group and its members? | I am not member of any association; To a high degree; To a modest degree; Not especially; Not at all; Don't know; No response; Several options checked |
| *Sense of affinity with relatives* | |
| Do you feel a strong sense of kinship with your relatives (beyond spouse/partner/children)? | Have no family; To a high degree; To a modest degree; Not especially; Not at all; Don't know; No response; Several options checked |
| *Sense of affinity with residence area* | |
| Are you rooted in and feel a strong sense of kinship with your neighborhood? | To a high degree; To a certain degree; Not especially; Not at all; Don't know; No response; Several options checked |
| *Being part of a group of friends* | |
| Are you part of a group of friends/acquaintances who have something in common or do some activity together (e.g., play cards, listen to music, go on excursions, etc.)? | Yes; No; Don't know; No response; Several options checked |

Cronbach’s alpha: Social Connection 0.90; Social Support 0.88.
